# The essence of life

**DOI:** 10.1186/s13062-016-0150-5

**Published:** 2016-09-26

**Authors:** Wentao Ma

**Affiliations:** College of Life Sciences, Wuhan University, Wuhan, 430072 People’s Republic of China

**Keywords:** The definition of life, Darwinian entity, Self-sustained system, Origins of life, Bioinformation

## Abstract

**Abstract:**

Although biology has achieved great successes in recent years, we have not got a clear idea on “what is life?” Actually, as explained here, the main reason for this situation is that there are two completely distinct aspects for “life”, which are usually talked about together. Indeed, in respect to these two aspects: Darwinian evolution and self-sustaining, we must split the concept of life correspondingly, for example, by defining “life form” and “living entity”, separately. For life’s implementation (related to the two aspects) in nature, three mechanisms are crucial: the replication of DNA/RNA-like polymers by residue-pairing, the sequence-dependent folding of RNA/protein-like polymers engendering special functions, and the assembly of phospholipid-like amphiphiles forming vesicles. The notion “information” is significant for us to comprehend life phenomenon: the life form of a living entity can just be defined by its genetic information; Darwinian evolution is essentially an evolution of such information, transferred across generations. The in-depth analysis concerning the essence of life would improve our cognition in the whole field of biology, and may have a direct influence on its subfields like the origin of life, artificial life and astrobiology.

**Reviewers:**

This article was reviewed by Anthony Poole and Thomas Dandekar.

## Background

Accompany with the development of molecular biology, which was marked at its origin by the discovery of the double helix structure of DNA (in 1953) [1], we have gained a tremendous amount of knowledge about the “secret” of life [2]. However, ironically, we are still uncertain about the essence of life, which can be manifested by the fact that to date even a consensus on the definition of life cannot be reached [3, 4]. In fact, the essence of life represents one of the several long-standing fundamental concerns of ours over the whole field of the natural sciences. As a case in point, the question “what is life?” was adopted as the title of Schrödinger’s famous pamphlet [5] – the author was a physicist himself; and notably, it was published (in 1944) even about a decade before we began to dig into the “secret of life” (i.e., the rise of molecular biology).

## The two distinct aspects of the concept “life”

Why we cannot reach a common opinion on the definition of life, though there have been numerous relevant discussions, or disputations? Now, the reason is not that we still lack some knowledge concerning the life phenomenon or the mechanisms underlying, but that we have not realized there are two distinct aspects for the concept of life, which is just a problem of logic.

We want to define “life” because we feel it is obviously different from the “non-life background”. For example, life can “replicate”, generating offspring, which is called the feature of “reproduction” in terms of biology (note: an idea emphasizing the difference between the two terms will be mentioned below when we talk about the implementation of life). Related to this feature, the specificity of an offspring individual is by and large derived from its parent(s), which is known as the feature of “heredity”; but the specificity of an offspring individual is not completely derived from its parent(s), which is relevant to the feature of “variation”. Perhaps more impressively, all living things we observe nowadays are leading, in its environment, a life style rather in favor of their survival and reproduction, which is referred to as the feature of “adaption”. All the features mentioned so far point to one essential aspect of life phenomenon: Darwinian evolution. “Reproduction”, manifesting “heredity” and “variation”, is the prerequisite of Darwinian evolution, whereas “adaption” is the result of Darwinian evolution.

But it isn’t over. Life is obviously different from its non-life background also in another aspect. It appears that any organism nowadays is a quite complicated system that “self-sustains”, involving energy and matter exchange with its environment. For example, a plant can absorb light energy and raw materials, and an animal may feed on plants to gain energy and raw materials it needs. Those biochemical reactions exploiting the energy and materials within the organism (to synthesize its own components), as a whole, is termed “metabolism”. Finally, “waste products” derived from the metabolism would be eliminated into the environment. If the organism “dies”, all these events, associated with the self-sustainment, would no longer occur. Concerning the wording “self-”, we should annotate a little more. Indeed, in accordance with the second law of thermodynamics, by the energy and matter exchange with its environment a living system can be sustained (or say, the order of the system can be kept) — this seems to be a feature of living systems. However, there are certainly also other open systems that are sustained in order, termed “dissipative systems”, such as Rayleigh–Bénard convection, Belousov-Zhabotinskii reaction, turbulence, cyclone, etc. The distinctive characteristic of a living system lies in that it is “self-sustained”, which means the energy-matter-exchange and the biochemical reactions involved in the metabolism are active events, depending upon the system’s own “functional components” (e.g., across-membrane transporters and enzymes).

Of course, the two aspects are associated with each other. For contemporary life, the functional components depending on which a living system can self-sustain are typically made of proteins (for possible primordial life in the hypothetic “RNA world”, the functional components were made of RNA [6–9]). No doubt, it is just Darwinian evolution that has given rise to these functional components. In fact, we may explain the whole tendency concerning such an evolution in a general way. Energy and raw materials are always the targets of competition in Darwinian evolution, thus always being in shortage. When a variation occurs resulting in the appearance of a function which enables the system to make use of more “fundamental” energy and raw materials (which are abundant), the variation would be maintained by natural selection. With the “in-depth” proceeding of such a tendency, more and more relevant functions would emerge, e.g., those catalytic functions might be organized into “metabolic pathways” finally. Thus, the system would become more and more complex and look increasingly “self-sustaining” – it, for instance, may ultimately be able to exploit rather “fundamental” energy such as sunlight and rather “fundamental” materials such as water, carbon dioxide and minerals. Of course, beyond the tendency to exploit more fundamental energy and material, which gives rise to anabolism, a living system may also develop the ability to exploit the “ready-made” energy and materials by feeding on other living systems, which brings about catabolism. Indeed, we could say that the self-sustainment is actually one manifestation of the life’s feature “adaptation”. That is, the second aspect that makes life distinctive – “self-sustaining”, is actually a result of the first aspect that makes life distinctive – “Darwinian evolution”.

Remarkably, a relatively popular definition of life, which was phrased by NASA, serving as a “working definition” for searching extraterrestrial life, says: “Life is a self-sustaining chemical system capable of undergoing Darwinian evolution” [10–12]. It is popular perhaps because it catches both the two aspects of life. However, the definition is essentially defective (which reflects well a common confused understanding on the concept of life). The people conceiving this expression did not realize that the two aspects are completely different from each other – so different that they even cannot be described in the same context. How can an individual system undergo Darwinian evolution? Darwinian evolution makes sense only for a lineage (from the level of population to that of species and that above). Or rather, Darwinian evolution does not refer to the evolution of an entity, but to the evolution of the form of that entity. In fact, if we accept the meaning of “evolution” as “becoming different over time”, we get to know that this kind of “evolution in form”, involving replication (reproduction) of individuals, means the descendant individuals become different from their ancestral individuals, rather than the alteration of a living individual itself. That is to say, in accordance with the two distinctly different aspects of the concept of life, the definition should split, as some expression like: “A life form is a matter form capable of undergoing Darwinian evolution; a living entity is a self-sustaining chemical system – in nature, it results from the Darwinian evolution and might engage into further Darwinian evolution”. In this definition, the word “life” is associated with the aspect of “Darwinian evolution”, and is in regard to a “form”; the word “living” is associated with the aspect of “self-sustaining”, and is in regard to an “entity”. See Table 1 for several examples concerning the application of such a definition.

**Table 1:** Several examples illustrating the “splitting” definition of the life concept

First, do viruses belong to life? This is a classic question reflecting our blurry concept concerning life. Obviously, a virus is not a self-sustaining chemical system – e.g., no metabolism when outside a host cell; however, when you say it is not life, you may look back and feel that it is indeed something quite different from the non-life background. Here we can make it clearer: the “form” of a virus is capable of undergoing Darwinian evolution, thus being a life form; a virus itself, as an individual, does not constitute a living entity (we may call it a “life entity” instead, see text). Second, is an old rabbit, no longer fertile, belongs to life? This is another classic question reflecting our blurry concept concerning life. Obviously, such an old rabbit no longer participates in Darwinian evolution, which characterizes life, but also obviously, it has not died – it is something quite different from the non-life background (surely, most of us would like to say it is alive). Here we can make it clearer: the form of the rabbit is no doubt capable of undergoing Darwinian evolution, thus being a life form; the old rabbit is no doubt still a living entity that self-sustains – though it will no longer engage into further Darwinian evolution. Note that as mentioned in the expression, a living entity results from Darwinian evolution, but, as an individual, only “might” engage into further Darwinian evolution. Third, somewhat oddly, even if a rabbit is a fertile one, there could still be doubts on whether it belongs to life because neither a male rabbit nor a female rabbit alone can perform reproduction [24], and how can it “undergo Darwinian evolution”? This is a more convincing example – that is, not only the incapability of reproducing, but also the incapability of performing reproduction alone – which is ordinary in the ubiquitous sexual reproduction, can cast doubt on the notion of confusing a living entity with its life form. When talking about Darwinian evolution, what we refer to is the “life form”. Indeed, if we do want to relate an entity with Darwinian evolution, we had better use the wording like “engaging into” instead of “undergoing”.	

In addition, there is a supplementary note to the definition. Logically, there should be an “intermediate” concept between the “life form” and the “living entity”. That is the entity which carries a life form, or say, the entity with a form capable of undergoing Darwinian evolution. We may call it a “life entity”, or a “Darwinian entity”. When we use the latter name, we emphasize that such an entity might engage in Darwinian evolution. By using the former name, we can realize directly the difference and relationship between the words “life” and “living” adopted here – conceptually, a living entity is just a complex life entity, in which the character of “self-sustaining” is developed, as explained above. Certainly, in practice, most life entities could be viewed as living entities because they should be more or less “self-sustaining”, except for those appearing in the very beginning of life (some supposition about relevant situations will be mentioned below), as well as those extremely simple parasites like viruses (see Table 1).

Certainly, given that “self-sustaining” has a blurry sense and the feature is derived from Darwinian evolution in nature, one that cannot endure a complex definition may be more satisfied with the definition of life with an emphasis on Darwinian evolution. That is, concisely, we may just define the “life form” mentioned above as the concept “life” itself: “Life is the form capable of undergoing Darwinian evolution” – but surely, one must bear in mind that, in this simplified definition,“self-sustaining”, a feature concerning “living” in our common sense, has not been reflected.

## Three key chemical mechanisms supporting the implementation of life

Above we have interpreted the concept of life according to its two distinctive aspects. However, under the topic concerning “the essence of life”, it would be better to inquire into the mechanisms in nature that render the implementation of the two aspects possible.

How can an entity engage into Darwinian evolution? As already mentioned, “replication/reproduction”, manifesting the characteristics of “heredity” and “variation”, is the prerequisite of Darwinian evolution. Here we consider this point in more detail. The entity should be capable of replicating with some extent of variation, and the variation should be inheritable (which means it can be passed on to the following generations). In the contemporary living world, such a kind of replication is mainly rooted in the template-directed copying of DNA (except for in some viruses and viroids, RNA instead), and in the hypothetic RNA world [6–9], such a kind of replication is rooted in the template-directed copying of RNA. No matter how, the underlying chemical mechanism is the base-pairing between the monomers of nucleic acids, in which hydrogen bonds play a crucial role. Indeed, this mechanism of “modular replication” [13] (polymer-replicating through template-directed copying based on monomer-pairing) meets the requirements mentioned above regarding the variable replication and inheritable variation exactly. It seems to be a “magical mechanism” in nature that makes the implementation of life possible. To date, no other mechanism has been “found” or “envisioned” to be able to satisfy these requirements, although there have been some efforts attempting to realize this mechanism on other molecules, such as some polymers akin to the nucleic acids [7, 14], and even some special organic molecules designed ad hoc [15].

However, the mechanism of “modular replication” is not adequate for the implementation of Darwinian evolution. Even if an entity can replicate with inheritable variation based on this mechanism, no natural selection would occur merely due to this feature, because the selection acts on the level of “phenotype” rather than that of “genotype”. That is to say, there must be corresponding functions derived according to the nucleic acid sequenceand, relevantly, corresponding functional alterations stemmed from the variations of the sequence. In the contemporary living world, the functions are mainly carried by proteins translated from mRNAs, which are in turn transcribed from the genes carried by DNA (except for in some cases, functions are carried directly by “functional RNAs”, transcribed from DNA). In the hypothetic RNA world, all the functions were carried by RNA, the same material carrying genes [6–9]. No matter how, a key mechanism that ultimately makes such a shift toward phenotype possible is: a molecule of the functional polymer (protein or RNA), in fact, folds to its special structure with its special function “according to” its special sequence. For the folding of the proteins, the hydrophobic interaction plays a crucial role; for the folding of the RNAs, hydrogen bonds (leading to intra-chain base-pairings) play a crucial role. In addition, noticeably, for the implementation of the second aspect of life, this mechanism also deserves enormous credit – it is just such structure-based functional polymers that could support the “self-sustaining” (e.g., enzymes; or ribozymes for an “RNA-based organism”), no else. Indeed, this mechanism, i.e., the sequence-dependent folding of RNA/protein-like polymers, which brings about corresponding special functions, seems to be a second “magical mechanism” in nature that renders life possible to arise.

Moreover, there is a third mechanism that is also very important for the implementation of life. Indeed, considering RNA molecules might both implement the modular replication and fold to carry functions, they, as individual molecules, may have been the simplest, earliest “Darwinian entities” (i.e., “life entities”). For example, some RNA species that can benefit its own replication, such as the one catalyzing the RNA replication (i.e., an RNA replicase ribozyme) [7, 14] and/or the one catalyzing the synthesis of its building blocks (i.e., a nucleotide synthetase ribozyme) [7, 16], may have emerged, say, in the beginning of the RNA world. However, more advanced entities that can harvest multiple advantages simultaneously should represent the subsequent direction of evolution. In nature, typically one functional molecule bears only one function – the reason has been implied in the second mechanism explained above: a functional polymer folds to its special structure with its special function according to its special sequence. That means a more advanced entity should have to comprise multiple functional molecules (e.g., both the RNA replicase ribozyme and the nucleotide synthetase ribozyme), which cooperate with each other. Hence, there should be a mechanism to keep these different functional molecules sufficiently adjacent. In this regard, it seems that no mechanisms can work better than the one suggested by our modern living cells: encompassing the functional molecules within a lipid vesicle [17] – this mechanism limits the movement/dispersal of the cooperating functional molecules and meanwhile, perhaps equally importantly, does not limit the spatial movement/dispersal of the resulting entities as integral individuals (see Ref. [18] for a more detailed discussion on the so-called “protocells”). The formation of the vesicle, with its membrane composed of amphiphilic molecules such as fatty acids and phospholipids, is a natural process occurring in water, wherein, again, the hydrophobic interaction plays a key role. Notably, it has been suggested that while those RNA-like modular-replicating entities are called replicators, such more advanced, cell-like entities should be referred to as “reproducers” [13, 19]. So the process of the reproduction contains the replication of replicators within the vesicle and the subsequent division of that vesicle. From then on, at the level of individual occurs no replication but reproduction (see the next section for a remark about more complicated reproduction when life form became more complex). This attempt to conceptually distinguish reproduction from replication reflects well the significance of the “vesicle” mechanism in regard to the first aspect of life – enabling the most fundamental step of life form’s evolution toward complexity, from the molecular level to the level above molecule (see Table 2 for a supplemental annotation to this point). In addition, remarkably, we can also tell the great significance of this mechanism in regard to the second aspect of life – it naturally “defines” a closed, independent system, based on which the so-called “self-sustaining” could make sense; and on the other hand, the system is not absolutely closed, which allows the matter-energy-exchange that is critical to the sustainment ( according to the second law of thermodynamics). Indeed, this mechanism concerning the formation of lipid vesicles, which gives rise to cell-like entities, seems to be the third “magical mechanism” in nature that makes the emergence of life possible.

**Table 2:** Self-organization accounts for the implementation of life above the molecular level

In fact, in the living world, to be functional, the folding of single functional molecules is sometimes insufficient. The formation of molecular complexes, involving interaction between biomolecules, may be important, such as hemoglobin, ion channels, and more representatively, the ribosome. This process of complex-formation is by and large akin to the assembly of amphiphiles to form vesicles, both of which are typical cases of the so-called “self-organization”. According to the description in Wikipedia, “self-organization is a process where some form of overall order or coordination arises out of the local interactions between smaller component parts of an initially disordered system”. That is, in a more general sense, we may say that it is self-organization (not only the assembly of amphiphiles forming vesicles) that enables the implementation of life from the molecular level to the level of complex entity.	

Finally, it should be noted that we are not overstating when we say all these chemical mechanisms may ultimately be ascribed to the features of those building blocks available. Undoubtedly, the formation of vesicle is owing to the feature of the phospholipids-like molecules, i.e., “amphiphilicity”; as mentioned already, the implementation of modular replication should be attributed to the feature of nucleotides/deoxynucleotides; apparently, the implementation of functions in the functional polymers is also determined by the feature of corresponding building blocks – amino acids for proteins and nucleotides for RNA. Indeed, just because the types of amino acids are more than those of nucleotides and the chemical property of the amino acids are more active than that of nucleotides, proteins, rather than RNA, act as the main functional molecules in modern life. As a more straightforward contrast, it is just the additional 2’-OH in nucleotides that determines that RNA is more suitable to act as functional molecules but less suitable to act as template molecules than DNA.

## Understanding life in terms of information

As mentioned above, when we talk about the evolution of life, we do not mean the evolution of individual entities but the evolution of their life forms. Actually, we can interpret such an “evolution in form” this way: a living entity bears and is characterized by its life form, and when it reproduces, it passes its life form on to its offspring; the life form evolves over generations, mainly ruled by the mechanism of Darwinian selection (more rigorously, here we should talk about “life entities vs. their life forms”, as explained in the section above concerning their definitions). See Table 3 for an example illustrating the significance of this notion. Indeed, to comprehend the characteristic of the life phenomenon, it is very important to clearly distinguish the form of an entity from the entity itself. Here, we can talk about this issue in more depth.

**Table 3:** The evolution of life is a sort of “evolution in form” which has nothing to do with thermodynamics

In people’s efforts to understand life phenomenon, there has long been a puzzle: “why can life evolve towards higher order and complexity, seeming to run against the second law of thermodynamics?” Certainly, the evolution of an individual living entity cannot disobey the second law of thermodynamics – for example, to be able to keep its own order (i.e., self-sustain), a living entity must be an open system, as mentioned already. However, rather than the evolution of individual entities themselves, the evolution of life means the evolution of their life forms, having nothing to do with the thermodynamic law. Indeed, as interpreted in the text, when a living entity reproduces, it transfers its life form to its offspring; the life form evolves over generations, through Darwinian selection, tending towards higher order and complexity. As a contrast, a non-life matter cannot reproduce, and thus its form cannot be separated from the entity – the form’s evolution is just identical to the entity’s evolution, which cannot escape the thermodynamic law.	

Let us start with the case of molecules. In regard of “matter”, two molecules, as independent entities, are certainly different from each other. But we also often say two molecules are of the same “kind”, e.g., two water molecules. Notably, the main aim of the science chemistry is just to explore the interactions/reactions between various “kinds” of molecules. In fact, generally speaking, the “kind” of a molecule just means the form of the molecule. This is easy to understand. Remarkably, there is a sort of special molecules in nature – the polymers constructed by monomer residues of several or more distinct types. Admittedly, when we talk about these polymers, we have in our minds those central biomolecules – DNA, RNA and proteins, or something alike. For such molecules, their forms are “sensitively” affected by their residue sequences – even the variation of a single residue at a certain locus can bring about molecules of different kinds. Actually, the form of such a polymer can be defined by the form of its residues and the sequence of the residues. Here it is notable that the sequence, manifesting as a succession of different “letters”, is just a typical “format” of “information”. That is, “information”, as a “quantifiable” notion in some way, may help us to define the “kinds” or “forms” of these “sensitive” polymers. We can say, for instance, that the form of a DNA molecule is just specified, or represented, by the information carried on its sequence (here we have predefined the molecule as DNA, so the form of its residues, i.e., nucleotide, is definite and needs not to be included in the statement).

Now we turn to the case of living entities. A living entity tends to be a complex system composed of many molecules (especially if we do not consider the situation in the very beginning of life as mentioned above). Likewise, two of such entities are certainly different in regard of matter because they are independent of each other. And similarly, it appears that they can also be of the same “kind”, e.g., two individuals of the same species. However, typically, two living entities cannot be completely identical in form, unlike two molecules in the chemical world. Indeed, the differences within the same species have accumulated generation by generation. Then, what is the essence of the accumulated differences? With little dispute, we can boil it down to the sequence of genomic DNA (or RNA sometimes), or say, as explained above, the information carried on this “sensitive” polymer. Surely, it is just the “genetic information” in terms of biology. In fact, due to some causes (e.g., the environmental isolation), the differences of the genetic information within a species may increase further, ultimately giving rise to different species – this is just the manner how the whole living world has arisen and is still evolving forward. In other words, we can say that the form of a living entity is in practice defined by its genetic information – it is this information that ultimately determines all the specificity of the living entity. Indeed, as somewhat few exceptional cases, two or more living entities might as well be “identical in form” – and this is just because their genetic information is “identical”, e.g., monozygotic twins (or multiples) and those clone-individuals.

Here we have traced the form of living entities into the form of, thus the information carried on, their genetic molecules (the so-called “sensitive” polymers, DNA or RNA). The genetic information is transferred across generations by the modular replication, and transferred within a generation via expression into the functional molecules (also the so-called “sensitive” polymers, proteins or RNA) – that is just what the Central Dogma tell us. Then, it is natural selection that closes the circle – acting on the phenotypes “figured” by the functional molecules and gives rise to further genetic information deserving transferring across generations (a more detailed explanation on this point will appear below). Additionally, see Table 4 for an interesting remark on the “strategy” of using the genetic information (to transfer life form), in regard to the emergence of complex living entities.

**Table 4:** Transferring life form by genetic information is significant especially when life become complex

For the kind of “form evolution” like that in the living world, there should have been an inevitable problem: how can the form of a complex system be passed on to the next generation? This seems not to be a problem that can be easily solved in nature – and it appears that no other strategy can substitute the strategy that is used by extant organisms: record the whole form of the system as genetic information and pass the information (by that magical modular replication via residue-pairing) on to the offspring, wherein the (phenotypic) form is “rebuilt” according to the genetic information. Notably, in line with the idea concerning the evolution of “reproducers” [13 19], when the life form became more complex (no longer being protocells as mentioned above), the replication of replicators (genes) within the entity would have become insufficient to “prepare for” the multiplication of individuals – a process called “development” would be introduced into the process of reproduction. Apparently, the “development” process fits well in concept with the “rebuilding” process mentioned here. Indeed, the more complex the life form is, the more complicated the development process would become. That is, in some sense, it is the adoption of this strategy, i.e., using the genetic information to transfer the life form across generations but relying on development to implement a living entity within one generation, that makes the emergence of complex living entities (indeed, rather complex) feasible.	

In fact, “information” is in itself a concept without a clear definition, and the reason seems to be similar to that for the concept of life – there are two distinct aspects for “information”: first, it represents something different from others (or say, background); second, it makes meaning someway. To make the situation more confusing, the meaning of information is dispensable in some context – indeed, as noted in the classic document concerning information communication [20], when information is transferred, only the specificity represented by the information is important. Nonetheless, genetic information in life could find its explanation with regard to the concept “information” in such a context. Firstly, the sequence of genomic DNA/RNA distinguishes the entity from others clearly. Secondly, the sequence makes meaning in respect that it may be expressed into proteins/RNA, functioning in favor of the entity’s survival/reproduction (in short, “adaption”). Thirdly, not all the sequence in the genome makes meaning – surely, there are nonsense areas, especially in eukaryotes; the sequence in these areas, though meaningless, does contribute to the identification of the entity from others; and no doubt, when the genetic information is transferred to the next generation (by the modular replication of genomic DNA/RNA), whether it has meaning or not is unimportant.

All in all, we can understand that the “evolution in form” concerning life (i.e., Darwinian evolution) is actually an evolution of information. In fact, Darwinian evolution constitutes a natural way to generate information: first, a mutation during the replication of the genome gives rise to a distinction; then, the distinction, favoring the living entity’s survival/reproduction or not, is subject to natural selection; if the result is positive, the new information would be “fixed” into the genome, maintained across generations. More explicitly, for instance, the natural selection can work this way: supposed that a residue at a position of the genomic DNA turns out to be “A”, rather than “T”, “C”, or “G”, and this specificity, by manifesting as phenotype, provides this individual with some advantages in “adaption”; then, this letter at this position may be maintained in the genome generation after generation. Indeed, as it is understood, selection resolves “uncertainty” and thus generates information [20] – in the living world, it is natural selection that generates genetic information. This is by and large right, except for a notation that if the mutation is “neutral”, the specificity may also be maintained in the genome across generations until the position mutates again – such “provisional genetic information” is most likely to occur in the nonsense areas of the genome mentioned above. No matter how, we can make a general statement that it is mainly natural selection, performing over numerous generations, that leads to the accumulation of genetic information in the genome of living individuals, resulting in our prosperous living world comprising numerous life forms.

Noticeably, it is just such a special relation between life and information that constitutes the root of that distinctive field: “bioinformatics”. No “chemo-informatics” or “physico-informatics” is comparable.

## Conclusions

“Life” is a concept with two completely distinct aspects: Darwinian evolution and self-sustaining. An effort to define “life” should describe the two aspects separately, as some expression like: “A life form is a matter form capable of undergoing Darwinian evolution; a living entity is a self-sustaining chemical system – in nature, it results from the Darwinian evolution and might engage into further Darwinian evolution”. Alternatively, on account of that “self-sustaining” has a blurry sense and it is derived from Darwinian evolution in nature, one that pursue a clear and concise definition may be more satisfied with a statement only emphasizing Darwinian evolution, like: “Life is the form capable of undergoing Darwinian evolution”.

Regarding its implementation in nature, life seems to be a miracle, owing to three magical chemical mechanisms (to realize its two aspects): the replication of DNA/RNA-like polymers by residue-pairing, the sequence-dependent folding of RNA/protein-like polymers engendering special functions, and the assembly of phospholipid-like amphiphiles forming vesicles.

The life form of a living entity can be defined in terms of information – that is, its genetic information; natural selection is the key to give rise to such information, which has accumulated over numerous generations.

The dissection about the essence of life would improve our cognition on the whole discipline of biology, “deepening” its “success” nowadays. More generally, it may promote our fundamental understanding on natural phenomena further. More particularly, it may have a direct influence on the fields like the origin of life, artificial life and astrobiology.

## Reviewers’ comments

I am grateful to the reviewers for their thoughtful analysis and comments on the manuscript. I think that the topic of this manuscript, which addresses a long-standing, controversial issue with respect to the most fundamental concept in the field of biology, is very suitable to appear in this journal, which has a policy to publish reviewers’ comments and authors’ responses together with the manuscript. I hope that the paper would aid in improving the situation that we, as researchers in the field of life sciences, unfortunately, are still hesitating about what on earth is “life”.

### Reviewer 1: Anthony Poole, Stockholm University, Sweden

## Reviewer comments

I am fine with the main argument. However, it is not new to the literature, and is not as crisply delivered as previous treatises, including some which the author cites.

Author’s response: *It is fine that the reviewer endorses the main argument of the paper. As for the novel contents of the paper, it will be shown in my following responses that the paper does tell something new to the literature.*

## Reviewer comments

This philosophical piece by Wentao Ma is a discussion of the definition of life. Ma covers well-trodden territory, arguing primarily for a Darwinian definition of life.

Author’s response: *Indeed, an important aspect concerning the discussion about the essence of life is to provide a clear definition of life, as it has been done here (see the section “The two distinct aspects of the concept ‘life’”). Just considering the awkward situation that not even a clear definition of “life” can appear in any textbook of biology, such an effort is of great significance. However, that is not all. Even when we can define the concept life in one or several sentences, e.g., using terms like “Darwinian evolution” and “self-sustaining”, we may still wonder about how such processes or features can be implemented in nature. This concern, turning from the “conceptual basis” to the “material basis” of life in nature, should also be included into the topic of “the essence of life” (see the section “Three key chemical mechanisms supporting the implementation of life”). Finally, once we can discern the form of life from living entities, and be aware of the material (chemical) basis of life – particular in regard of those biopolymers, we will comprehend why “life” is deeply associated with another fundamental concept in nature, “information”; and this comprehension, in turn, will no doubt promote our understanding of life phenomenon (see the section “Understanding life in terms of information”).*

*In addition, though it is attractive to endorse a pure Darwinian definition of life, as explained when I introduce the alternative definition “Life is the form capable of undergoing Darwinian evolution” in this paper, here it should be noted that the availability of “self-sustaining” is rather important regarding the outcome of Darwinian evolution, as we can see in our modern living world. Without this aspect, the life forms would have stayed at a rather simply level, perhaps too trivial to be distinguishable from the background non-living world. Such an understanding may be useful for our efforts in the field of astrobiology.*

## Reviewer comments

While I don’t particularly disagree with the main points, the paper really doesn’t cover any new ground, and in my view, other papers do a better job of dealing with this topic. For instance, Szathmáry makes a very helpful distinction between replicators and reproducers [19]. In contrast, Ma uses the terms replication and reproduction interchangeably. This is not helpful and serves to blur an important distinction between these. The point made by Ma, that life requires three ‘mechanisms’ (replication of a template, generation of functional molecules, and a physical boundary) is fine, but not new, and does not advance current thinking. This is nicely articulated in Ganti’s chemoton model, which consists of three parts (metabolic system, system for heritable control, a boundary system) [21]. Importantly, the chemoton model led to the concept of infrabiological systems (see [19]), which lack one of these three components, and is a helpful formulation for understanding the emergence of life. Mix [22] provides clearer categorical definitions than Ma, who is primarily interested in Darwinian evolution. Ma draws a distinction between ‘life form’ and ‘living entity’, where the first seems to be ‘Darwin life’ and the second is ‘Haldane life’ by Mix’s definitions. Note that Mix provides a third definition that neatly enables us to categorise all cellular life as ‘Woese life’, and also notes that multiple definitions may be applied to some entities. Finally, it is unfortunate that Ma uses the term ‘magic’ in several places - this is both vague and unhelpful. I would encourage the author to take the time to rethink their article with reference to the works [19, 21, 22] (as well as those cited within these key works).

Author’s response: *Thank to this journal’s policy that publishes reviewers’ comments and authors’ responses, and also thank to this reviewer’s careful remarks, here we have the chance to discuss these interesting issues related to the manuscript.*

*Firstly, I have noticed the efforts to make a distinction between replicators and reproducers [*19*] (earlier in [*13*]). As explained by Szathmáry and his coworkers, new individuals of replicators arise by copying, whereas those of reproducers do not. Life may have originated as replicators (e.g., some RNA species) and evolved into reproducers when they were encapsulated by vesicles (e.g., RNA-based protocells). Then the process of reproduction should contain the replication of replicators within the vesicle and the subsequent division of that vesicle. When the “organisms” became more complex, the process of reproduction would become more complicated, tied with the process of “development”. In the original version of the manuscript, to be more focused, I did not mention this idea. But after rethinking the manuscript with reference with this idea (as suggested by the reviewer), I find that this idea is in fact fairly related to our topic (i.e., “the essence of life”) – with respect to how life, especially those complex life forms, can be implemented in nature. So I have mentioned this idea and added relevant remarks in the new version (see the penultimate paragraph of the section “Three key chemical mechanisms supporting the implementation of life” and* Table 4*in the section “Understanding life in terms of information”). Thank the reviewer very much for his kind suggestion.*

*Secondly, I have also noticed Ganti’s chemoton model [*21*], which has been discussed in detail in Szathmáry’s papers mentioned above [*13*,*^19^*]. In my opinion, the model is more like an “empirical model”, which is only summarized from the life phenomena we can observe in appearance. That is, it does not reflect why the three parts (metabolic system, system for heritable control, a boundary system) are essentially required. On the contrary, here I start from the two fundamental aspects of life (Darwinian evolution and self-sustaining) and explain the essential roles of the three “mechanisms” (replication of a template, generation of functional molecules, and a physical boundary). Tracing into the conceptual basis and the material basis of life is just one important novelty of the present paper. Noticeably, just owing to this distinction of the starting point, we can see an important difference between these two ideas. “Generation of functional molecules” is not completely paralleled with “metabolic system” – in fact, only a portion of the functional molecules were involved in metabolism, which supports the self-sustaining. This is apparent in modern life, e.g., numerous proteins serves as structural blocks, rather than enzymes. Indeed, as indicated in this paper, self-sustaining is only one (not all) outcome of Darwinian evolution. Also due to such a standpoint, regarding the infrabiological systems, I tend to think that heritable control is indispensible – that is, an infrabiological “metabolic + boundary” system during the emergence of life is impossible. Additionally, here we can see that the Ganti’s chemoton model, as a typical idea in the field of the origin of life, attempts to summarize the sense of Darwinian evolution and self-sustaining in the same context, without discerning life form from living entities. Such a situation has brought about quite a lot of confusions and controversies in the field. The attempt to rectify this situation just represents the most important novelty of this paper.*

*Thirdly, I thank the reviewer for bringing the paper [*22*] to my attention. In this paper, initially, Mix criticized the recently “popular” viewpoint that definitions of life are impossible or impractical. I endorse the author’s opinion completely. Then it was suggested that since it is difficult to reach a consensus on the definition, we should pursue provisional definitions for clear communications. This also seems to be a correct attitude, because we need to tell other people clearly what we mean when we mention the word “life” (especially in the fields of the origin of life, artificial life and astrobiology). First, he suggested the life closed to modern cellular life can be referred to as Woese life, owing to Woese’s original work that related them by similarities in their rRNA. In my opinion, this summarization, like the chemoton model, is directly phenomenon-based, at least unhelpful for us to envision the essence of life. Then, he introduced “Darwin life – exhibiting evolution by natural selection” and “Haldane life – exhibiting metabolism and maintenance”. Apparently, the author has recognized that there are two completely distinct aspects for life, but he did not associate this recognition with the essence of life. As he wrote in the paper, “Our categories need not be essential or substantial, only methodologically useful” – “Woese life, Darwin life, and Haldane life” are only used to “represent clear categories about which we can make unambiguous statements without committing to whether they are ‘life’ in any larger sense”. More importantly, the author and all other people with similar insights (see the paper and references therein for details) failed to realize the distinction of “form” and “entity” in relation to the life phenomenon. That is, Darwin life should be actually in respect of the “kinds”, i.e., the life forms, whereas Haldane life should refer to individuals, i.e., living entities. Indeed, I would like to say to Mix that bringing his understanding of the distinction between “Darwin life” and “Haldane life” to the level of the essence of life, the provisional definitions may turn into an ultimate one.*

*Finally, as to the term “magic”, I want to express the idea that it is far from an easy thing to implement life in nature – in regard to the two aspects of life, Darwinian evolution and self-sustaining (that is, it is nearly a miracle). The idea is helpful for us to understand that why we can find only one type of life on the earth, i.e., the one materially based on DNA/RNA/proteins and lipid vesicles, which is often referred to as “life as we know it”. Moreover, this annotation should also aid in our efforts to search “extraterrestrial life”. This seems to be out of question. So I guess the reviewer means that the noun “magic” which is used to refer to the three key chemical mechanisms is not suitable. Therefore, I have changed the noun “magic” to the phrase “magical mechanism” in relevant places.*

### Reviewer 2: Thomas Dandekar, Department of Bioinformatics, University of Wuerzburg, Germany

## Reviewer comments

First of all, why am I reviewing this manuscript so fast? Well, there is nothing more important than the essence of life, so I have also time to review this. However, working on this topic and getting non-trivial results is far from easy. My impression is that in this article we have a clear, non-trivial distinction between two processes essential for life: (i) Darwinian Evolution versus (ii) the self-sustaining capability of life. The author nicely points out that a distinction of both central features of life leads to further insights. I liked the article, it is thoughtful and so I added some thoughts on it and hope they will improve the already nice piece of work.

Author’s response: *Yes, the reviewer’s interpretation of the central idea of this manuscript is almost perfect. Many thanks for the reviewer’s warm comments.*

## Reviewer comments

I have the following helpful comments for this nice commentary: The author should incorporate some other perspectives on this. This can easily be done, few sentences are enough as the insightful commentary should be kept short, to make nice reading: a) Explain the notion of information better, as this interestingly always depends on who reads this information. So here, as rightly stated, this information arises by selection and so this type of information is only possible to come about by feature (i) active evolution (more information on this including some insights on how “meaning” develops by such processes as subjective phenomenon can be found in https://opus.bibliothek.uni-wuerzburg.de/frontdoor/index/index/docId/2749).

Author’s response: *“Information” is another concept without a clear definition, the reason of which seems also to be that it contains two distinct aspects: first, it represents something distinct from others; second, it makes meaning someway. To make things worse, as mentioned in the classic document of information communication [*20*], the second aspect, i.e., the meaning of information, is sometimes dispensable. In the manuscript, to be more focus and avoid unnecessary controversy in this respect, I, deliberately, did not explain the notion of information in detail. However, as noticed by the reviewer, it seems hard to evade this point under the explicit subtitle “Understanding life in terms of information”. So I have tried to talk about these fundamental points of “information” and associate them with genetic information and Darwinian evolution in the new version (see the antepenultimate paragraph and the penultimate paragraph of this section). Certainly, readers may see the web site provided by the reviewer for a more extensive discussion on relevant topics. Thank the reviewer for his kind suggestion.*

## Reviewer comments

b) The “magical chemical process”: Yes, rightly observed. This happens as there is also the feature (iii) of self-organizing processes. Only in a world where such processes exist in sufficient extent life is possible and such things as the three magical chemical processes come about. So please mention this basic feature of a world in which life is possible. It would be fair to mention here Stephen Jay Gould who spoke about “the exaptive excellence of spandrels…” to explain the power of building blocks [23]. So, for such magic to happen, suitable building blocks are necessary, they determine which “game” can be played by evolution (insight by Stephen Jay Gould) - in fact seriously limiting the play and its contents, for instance our life will be relying on DNA as information storage and that limits certain “games” we can achieve by our evolutionary processes. My contention would be that the building blocks have to be with some self-organizing capabilities (as amply shown also by other authors on this topic).

Author’s response: *Yes, I agree with the reviewer. So I add a table (*Table 2*) and a paragraph at the end of the section “Three key chemical mechanisms supporting the implementation of life” to mention these points. Note that, to be more focused, I have constrained the sense of “building blocks” – here only in regard to the three key chemical mechanisms mentioned in the text, i.e., nucleotides/deoxynucleotides, amino acids and phospholipid-like amphiphilic molecules.*

## Reviewer comments

c) If you follow the argument (“b”), the building blocks are so critical, you appreciate even more, that with human civilisation a new factor appears: you are sufficiently complex to leave the limitations of building blocks, in particular you can build machines and cars, you can establish artificial intelligence, so new technical processes, which do not have the limitation of the building blocks, and (showing the non-trivial insight of the article) these technical processes first improve feature (ii) self-sustaining capabilities of life (simple example: air conditioning when it is too hot in summer, achieved by a machine), and as the feature (ii) is critical for life and evolution of technical apparatus just started, we have currently problems arising from our technical civilisation and clearly, you can also investigate feature (i), evolutionary capabilities in technical processes, e.g. evolution of software, of computers, of cars etc. One can argue if you look at this, whether after all our building blocks are the key feature of life here on earth, to separate this type of life (according to the definitions of the author) from the features of our technology using other building blocks, but trying to fulfill also the two aspects of life discussed by the author but only starting to achieve this in a satisfactory way.

Author’s response: *I appreciate the reviewer’s interpretation on the significance of the building blocks in a general sense. I also admire the author’s marvelous extension of the meaning of the two key features of life. In particular, it is interesting to envision that “life form” can finally leave the limitation of building blocks in a chemical sense, blending into human civilisation – a real information-ruled world. These ideas are thoughtful but perhaps run beyond the scope of our current topic. Let us enjoy the policy that allows readers to see the reviewer’s idea along with the author’s.*

## Abbreviations

NASA: National Aeronautics and Space Administration
